# Genetic Algorithm for TMS Coil Position Optimization in Stroke Treatment

**DOI:** 10.3389/fpubh.2021.794167

**Published:** 2022-03-11

**Authors:** Shujie Lu, Haoyu Jiang, Chengwei Li, Baoyu Hong, Pu Zhang, Wenli Liu

**Affiliations:** ^1^Center for Medical Metrology, National Institute of Metrology, Beijing, China; ^2^China Academy of Telecommunications Technology, Beijing, China

**Keywords:** TMS, stroke, voxel of interest, genetic algorithm, coil position optimization

## Abstract

Transcranial magnetic stimulation (TMS), a non-invasive technique to stimulate human brain, has been widely used in stroke treatment for its capability of regulating synaptic plasticity and promoting cortical functional reconstruction. As shown in previous studies, the high electric field (E-field) intensity around the lesion helps in the recovery of brain function, thus the spatial location and angle of coil truly matter for the significant correlation with therapeutic effect of TMS. But, the error caused by coil placement in current clinical setting is still non-negligible and a more precise coil positioning method needs to be proposed. In this study, two kinds of real brain stroke models of ischemic stroke and hemorrhagic stroke were established by inserting relative lesions into three human head models. A coil position optimization algorithm, based on the genetic algorithm (GA), was developed to search the spatial location and rotation angle of the coil in four 4 × 4 cm search domains around the lesion. It maximized the average intensity of the E-field in the voxel of interest (VOI). In this way, maximum 17.48% higher E-field intensity than that of clinical TMS stimulation was obtained. Besides, our method also shows the potential to avoid unnecessary exposure to the non-target regions. The proposed algorithm was verified to provide an optimal position after nine iterations and displayed good robustness for coil location optimization between different stroke models. To conclude, the optimized spatial location and rotation angle of the coil for TMS stroke treatment could be obtained through our algorithm, reducing the intensity and duration of human electromagnetic exposure and presenting a significant therapeutic potential of TMS for stroke.

## Introduction

Stroke is a prevalent disease worldwide and has caused a heavy burden on the healthcare system (1–3). As a serious life-threatening disease, which is common in middle-aged and elderly people, stroke can cause sequelae such as hemiplegia and aphasia (4, 5). Strokes, which cutoff the blood supply to parts of the brain, are categorized as ischemic stroke and hemorrhagic stroke. Ischemic stroke is caused by a sudden reduction in blood perfusion or complete interruption of blood flow to the local brain tissue. Hemorrhagic stroke is caused by cerebral hemorrhage or subarachnoid hemorrhage (6). The cortical branches of the middle cerebral artery (MCA) extend to the functional areas of the cerebral cortex and provide 80% of the blood supply to the brain (7). Stroke in the main branches of the MCA can cause severe physical injury to the body.

Transcranial magnetic stimulation (TMS) is an effective and non-invasive neuromodulation and therapy technique, which gained popularity in scientific research and clinical applications (8). Transcranial magnetic stimulation device generates an electric current in coil by the discharge of capacitor, which will activate pulsed magnetic field in space. This magnetic field passes through the skin and other tissues to generate an eddy current electric field (E-field) in the intracranial tissue, consequently stimulating the targeted brain area and affecting neural activity (9, 10). TMS can accelerate the cortical blood supply to the targeted brain areas, effectively enhance synaptic plasticity and regulate excitability of nerve cells as well as release of neurotransmitters in short and long terms, thus enhance the stimulated network interaction (11–16). These above characteristics are of great significance for stroke rehabilitation.

During stroke rehabilitation, the existence of viable neurons at stimulation area of TMS highly contributes to the therapeutic effect. For small stroke lesions, functional recovery depends on the recruitment of peripheral or residual neurons (17). Murata et al. reported that the plasticity of neural activity, functional connectivity in the premotor cortex, and the remaining tissue near the lesion contribute to the functional recovery of induced motor defects following damage to the primary motor cortex. Therefore, direct promotion of affected brain area excitability could be more beneficial to poststroke recovery than inhibition of unaffected brain area excitability (18, 19) and the higher E-field intensity is, the more efficient therapy is. According to the International Federation of Clinical Neurophysiology, the scalp spot where the targeted muscle reaches the peak motor evoked potential is defined as a “hot spot,” which is widely used in clinical practice for TMS stimulation (13, 20, 21). However, the “hot spot” definition is only a rough trade-off under the existing conditions without considering the angle of coil. In addition, since every individual has different cortex structures and the cortex folds of different brain areas of specific person are not the same, there is a high chance that the potential optimal stimulus “hot spot” remains undetected. Therefore, the targeted region may not acquire significant activation under specific TMS frequency and intensity, whereas the cortical and subcortical distant brain areas are activated from time to time (22, 23).

The therapeutic effect of TMS depends on the coil position, stimulation frequency, current intensity, etc. A set of accurate coil location and rotation angle can provide the most potent stimulation to the targeted brain area, while minimizing the impact on unrelated brain areas, which is crucial to an enhanced therapeutic effect (24, 25). However, coil placement by TMS operators is often accompanied by a deviation of 2 cm (26), which may affect the treatment efficiency through the change of the E-field distribution in target region. Therefore, it is necessary to study a more accurate clinical quantitative treatment scheme and a stimulus dose plan for the safe and precise TMS treatment (27). From what has been discussed above, the primary aim is to stimulate a more precise “hot spot” to acquire the E-field as high as possible for a stronger stimulation effect, which is truly helpful for activating neurons around the lesion. Some studies established comprehensive brain atlas of stimulation sites for quick operations to improve TMS stimulation accuracy (28, 29), whereas others used several heuristic algorithms, such as the fast computational auxiliary dipole method (ADM) (30) and particle swarm optimization (PSO) (31, 32), to regulate the position or current configuration of the coil to improve the overall E-field in the voxel of interest (VOI); thus, achieving optimal neural regulation. However, both these coil location optimization algorithms are based on a healthy brain. It is unknown if the algorithms are robust in the brain area with local conductivity changes, which mean that the coil position optimization of TMS stimulation near the lesion needs further study.

In this study, a method for TMS coil positioning is suggested, which will help clinician to find the optimal stimulus spot and get the E-field intensity near the lesion as high as possible for a better therapeutic effect. The optimization algorithm is based on the precision digital human head model with a stroke module inserted to simulate the two most common clinical MCA strokes (ischemic stroke and hemorrhagic stroke). Compared with other TMS coil position optimization algorithms for healthy human head models, the advantages of our method are a broader search domain, more coil positioning patterns, and a higher calculation efficiency. Further, the E-field intensity of the VOI is kept high and in the meantime, the intensity of the non-target region is under control to void any unnecessary exposure due to the constant emission energy. This method will have a great significance in clinical stroke rehabilitation during TMS treatment. The introduction of the optimization algorithm into TMS can effectively improve the stimulation efficiency of the treatment, reduce human electromagnetic exposure, and alleviate the concern of the public about electromagnetic exposure.

## Materials and Methods

### Numerical Models

**Figure 1:**
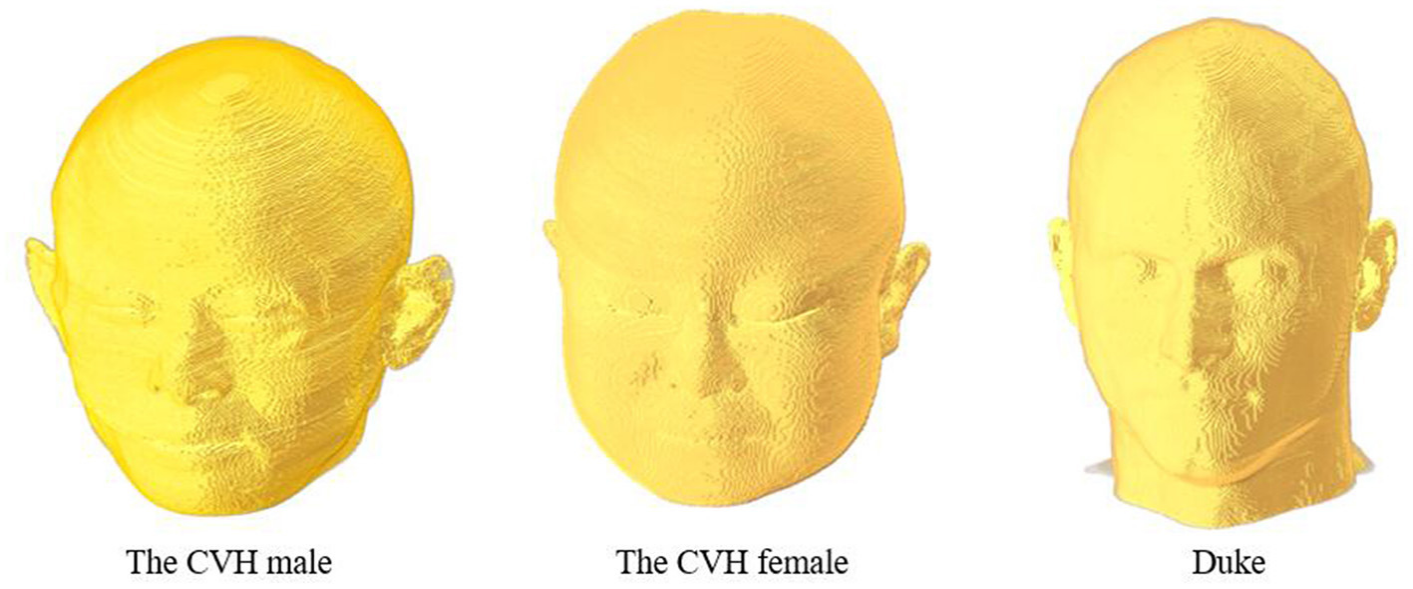
Numerical head model.

This study was based on three digital human head models (Figure 1) obtained from the Chinese Visible Human (CVH) project and the Virtual Family Project (33, 34). The two Chinese human head models included those of a 35-year-old male (CVH male) and a 22-year-old female (CVH female). The male and female models had resolutions of 1.0 × 1.0 × 1.0 and 0.5 × 0.5 × 0.5 mm, respectively. The third head model was that of a 34-year-old male Duke, with a resolution of 0.5 × 0.5 × 0.5 mm. The models contained more than 40 tissues, such as skin, skull, and fat, covering 20 to 35-year-old males and females belonging to the yellow and white races.

**Figure 2:**
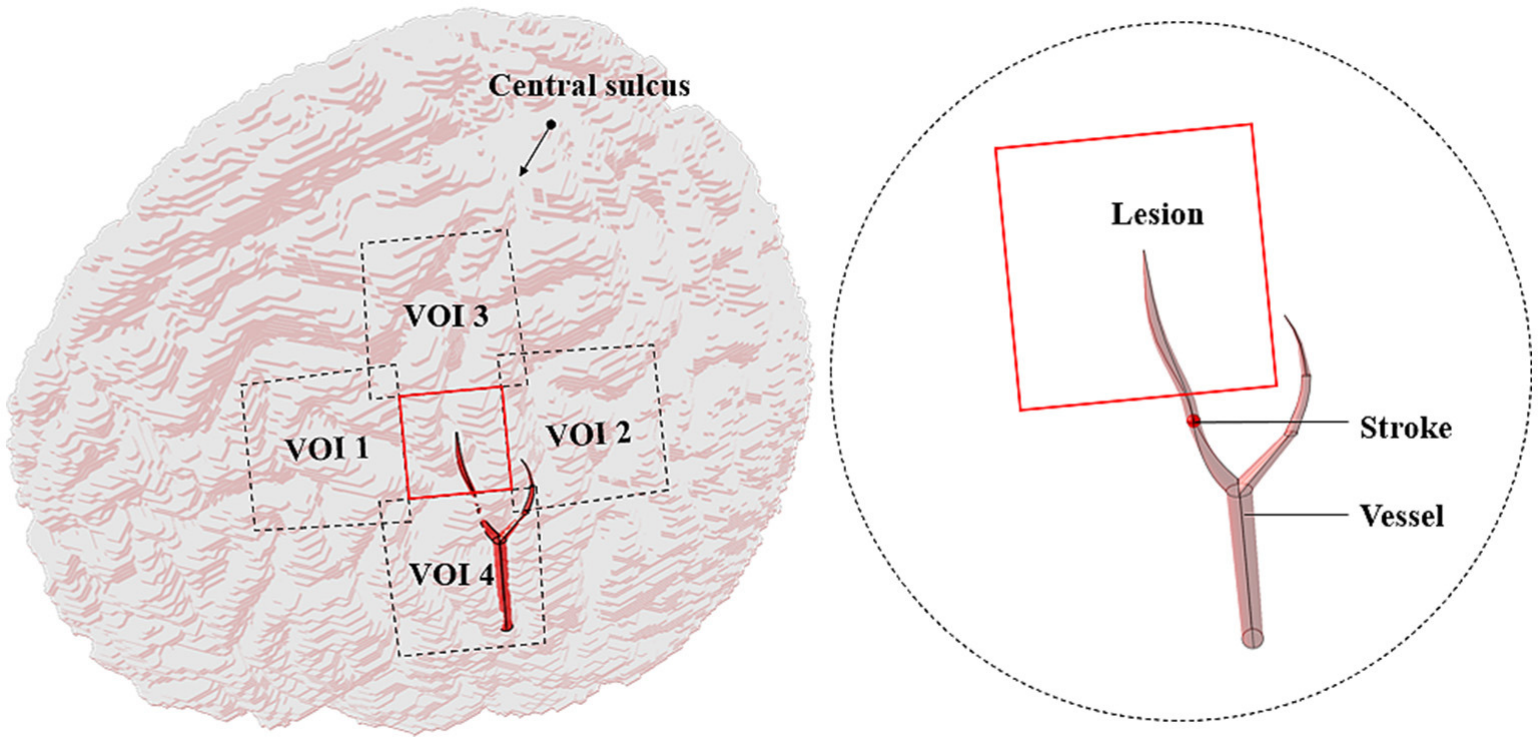
Stroke model (a part of vessels with stroke only).

The MCA disease is an important cause of stroke in humans (35). The central artery is the significant one of the MCA cortical branches (36). If this vessel suddenly ruptures or embolizes, some brain areas would suffer from pathological changes due to the lack of nutrients, resulting in temporary or permanent loss of brain function. To simulate the intracranial physiological state caused by stroke, a part of the central artery, thrombus, and the lesion (2 × 2 cm) with the four VOIs (3 × 3 cm) nearby were added to the cortex (Figure 2). The average diameters of the blood vessel and thrombus were both 1.9 mm (37, 38). The ischemic stroke and hemorrhagic stroke models were established by adjusting the tissue electromagnetic parameters of the lesion. When an ischemic stroke occurred due to blocking the cerebrovascular, the affected gray matter (GM) conductivity decreased by 10–14% (39). In addition, the complex dielectric constant value decreased by 10% (40). When a hemorrhagic stroke occurred, blood infiltrated the cortical tissue and completely replaced brain tissue, such that the GM conductivity of the lesions was the same as that of blood (41). Table 1 shows the tissues and all the electromagnetic parameters of models.

**Table 1:** Electromagnetic parameters of tissues (*f* = 2,240 Hz).

**Tissue**	**Conductivity (S/m)**	**Relative permittivity**
Skin	2.00E-04	1.14E+03
Cortical bone	2.03E-02	1.56E+03
Cancellous bone	8.19E-02	6.19E+03
Cerebrospinal fluid	2.00E+00	1.09E+02
Gray matter	1.04E-01	8.56E+04
White matter	6.42E-02	3.71E+04
Cerebellum	1.24E-01	8.59E+04
Hypophysis	5.26E-01	3.07E+04
Hypothalamus	1.04E-01	8.56E+04
Hippocampus	1.04E-01	8.56E+04
Fat	4.23E-02	7.47E+03
Pineal gland	5.26E-01	3.07E+04
Intervertebral discs	8.30E-01	6.07E+01
Spinal cord	3.02E-02	6.12E+04
Dura	5.01E-01	3.00E+03
Red bone marrow	1.02E-01	2.73E+03
Muscle	3.31E-01	1.44E+05
Cornea	4.25E-01	9.04E+04
Lens cortex	3.31E-01	4.71E+04
Nucleus	2.00E-01	9.91E+02
Iris	3.31E-01	1.44E+05
Sclera	5.07E-01	3.15E+04
Vitreous body	1.50E+00	9.90E+01
Retina	1.04E-01	8.56E+04
Aqueous humor	2.00E+00	1.09E+02
Lacrimal apparatus	2.00E+00	1.09E+02
Salivary gland	6.70E-01	9.16E+01
Respiratory tract	0.00E+00	1.00E+00
Tongue	2.76E-01	3.22E+04
Teeth	2.03E-02	1.56E+03
Nerve	3.02E-02	6.12E+04
Cartilage	1.75E-01	1.27E+04
Lymph node	5.90E-01	9.48E+01
Blood and stroke	7.00E-01	5.26E+03
Ischemic lesion	9.11E-02	7.71E+04
Hemorrhagic lesion	5.00E-01	5.26E+03

There exist numerous gyrus structures and specific cortical curvatures; thus, the lesions and the VOIs in different models have different thicknesses. The average thickness of lesions in the models ranged from 0.48 to 1.22 mm and that of the VOIs ranged from 1.74 to 4.67 mm (Table 2).

**Table 2:** Average thickness of the voxel of interests (VOIs) and lesions in stroke model (mm).

**Model**	**VOI 1**	**VOI 2**	**VOI 3**	**VOI 4**	**Lesion**
The CVH female	1.74	1.85	2.16	1.75	0.48
The CVH male	4.11	4.59	3.23	2.63	1.22
Duke	4.67	3.62	3.10	4.56	1.21

*The average thickness is determined by the ratio of volume (V) to projected area (S) (thickness = V/S)*.

### Numerical Simulation for the Induced E-Field

Transcranial magnetic stimulation was used to stimulate the area near the lesion to facilitate the function of residual neurons. A higher average value of the E-field intensity in the VOI resulted in a better stimulus effect. In TMS treatment of stroke, an eight-figure coil is often placed in a tangent position to the scalp and the handle of the coil is tilted to the rear of the midline at an angle of 45° (21, 42). At this time, the handle of the coil is perpendicular to the central sulcus and the induced current is perpendicular to the central sulcus, such that the induced E-field is significantly enhanced (43).

In the simulation, every model was divided into 1.0 × 1.0 × 1.0 mm voxels. Single-turn eight-figure coil with two wings of 70 mm diameter with currents in the opposite direction and no thickness was used as an excitation source (44). The frequency and intensity of the abstract harmonic current were 2.24 kHz and 1,000 A, respectively.

The magnetic vector *A* was introduced into the process of solving electromagnetic fields to calculate the induced magnetic field of the line current in the space using the Biot–Savart law.


(1) $$\begin{array}{l} A=\frac{\mu }{4\pi }\underset{V}{\int }\frac{J\left(x^{\prime }\right)dV}{r} \end{array}$$

Where, μ = μ_0_ is the magnetic permeability, *r* is the distance between the source element *x*′ and the model element *x*, and the source current is *J*(*x*′).

Combining Maxwell's equations, the current continuity equation derived from linear media gives:


(2) $$\begin{array}{l} \nabla \times \frac{1}{\mu }\nabla \times A=\omega^{2}\tilde{\varepsilon }A-j\omega \tilde{\varepsilon }\nabla \phi +J_{0} \end{array}$$

Where, $\tilde{\varepsilon }$ is the complex permittivity, ω is the angular frequency, *J*_0_ is the source current, and the scalar potential is ϕ.

Thereby, the E-field intensity can be obtained in each voxel:


(3) $$\begin{array}{l} E=-j\omega \text{A}-\nabla \phi =-j\omega A-\frac{\omega^{2}\tilde{\varepsilon }A+J_{0}-\nabla \times \frac{1}{\mu }\nabla \times A}{j\omega \tilde{\varepsilon }} \end{array}$$

### Genetic Algorithm

In the genetic algorithm (GA), a group of solutions (individuals) is obtained through several generations of evolution. In every generation, each individual codes in a specific way and the fitness function is used to evaluate its adaptability. Based on the principle of “survival of the fittest,” individuals with poor performance are eliminated and those individuals with excellent performance are selected to enter the next generation. The individuals are recombined under the influence of gene crossover and gene mutation to form a new genotype (45). Both the theory and experiment verify the robustness of the GA in a complex search space (46).

In order to identify the best position of the coil, the population of each generation consisted of 10 individuals and the genotype of an individual was determined by six bits of binary coding of their position *P*_*i*_ (*x*_*i*_, *y*_*i*_, *z*_*i*_, *and φ*_*i*_). The steps of *x*_*i*_ and *y*_*i*_ were both 1 mm and then *z*_*i*_ could be calculated according to the placement of the coil relative to the scalp. The step of the rotation angle φ_*i*_ was 2.8125°. It should be also mentioned that the *x*_*i*_, *y*_*i*_, *z*_*i*_, and φ_*i*_ were all in float format as a result of data code and decode process and were simplified to two decimal places further. The 24 bits of binary code could cover all the genotypes of individuals in the search domain. Each genotype corresponded to one phenotype (spatial location and rotation angle of the coil). The search domain covered *x* ∈ [−20 + *x*_0_, *x*_0_ + 20], *y* ∈ [−20 + *y*_0_, *y*_0_ + 20], where *x*_0_ and *y*_0_ are the central coordinates of the VOI. In each generation, individuals were involved in 0–1 mutation and randomized crossovers. The fitness value of each coil position was evaluated by the fitness function (the average E-field intensity in the VOI) (47). A higher average value of the E-field intensity in the VOI resulted in a better fitness value. Finally, natural selection was performed according to the fitness values; individuals with the worst fitness values were eliminated and those individuals with excellent fitness values were copied as offspring according to the probability. The number of iterations was set to 9. The algorithm idea was as follows:

**Begin:**

T ← 0; //T: Number of iterations
Initialize Pop(T); //Initial population
Fitness E(*x, y, z*, ϕ); //Survival of the fittest
**While** (**not** terminating number of iterations) **do**:
  Crossover operation to Pop(T);
  Mutation operation to Pop(T);
  T ← T + 1;
  **End**
  **Output:** Optimal fitness E(*x, y, z*, ϕ) and corresponding
  parameters (*x, y, z*, ϕ)

**End**

Following the idea of the GA, we wrote an algorithm to search the coil location and angle in the VOIs. The optimization algorithm controlled Sim4Life version 6.0 (ZMT Zurich MedTech AG, Zürich, Switzerland, UK) for the E-field distribution, solving the stimulation at random spots in the coil search domain. The optimal position of the coil with the peak VOI average E-field intensity was obtained to improve the effectiveness of magnetic stimulation during the treatment of stroke. The 2.5 GHz Intel I7–11700F 16-core processor was used for the calculation and the time of each simulation iteration was set to 2–3 min. The resolution was 1.0 × 1.0 × 1.0 mm and the convergence tolerance difference was 1.00E-08.

### Quantitative Evaluation Parameters of the E-Field

**Figure 3:**
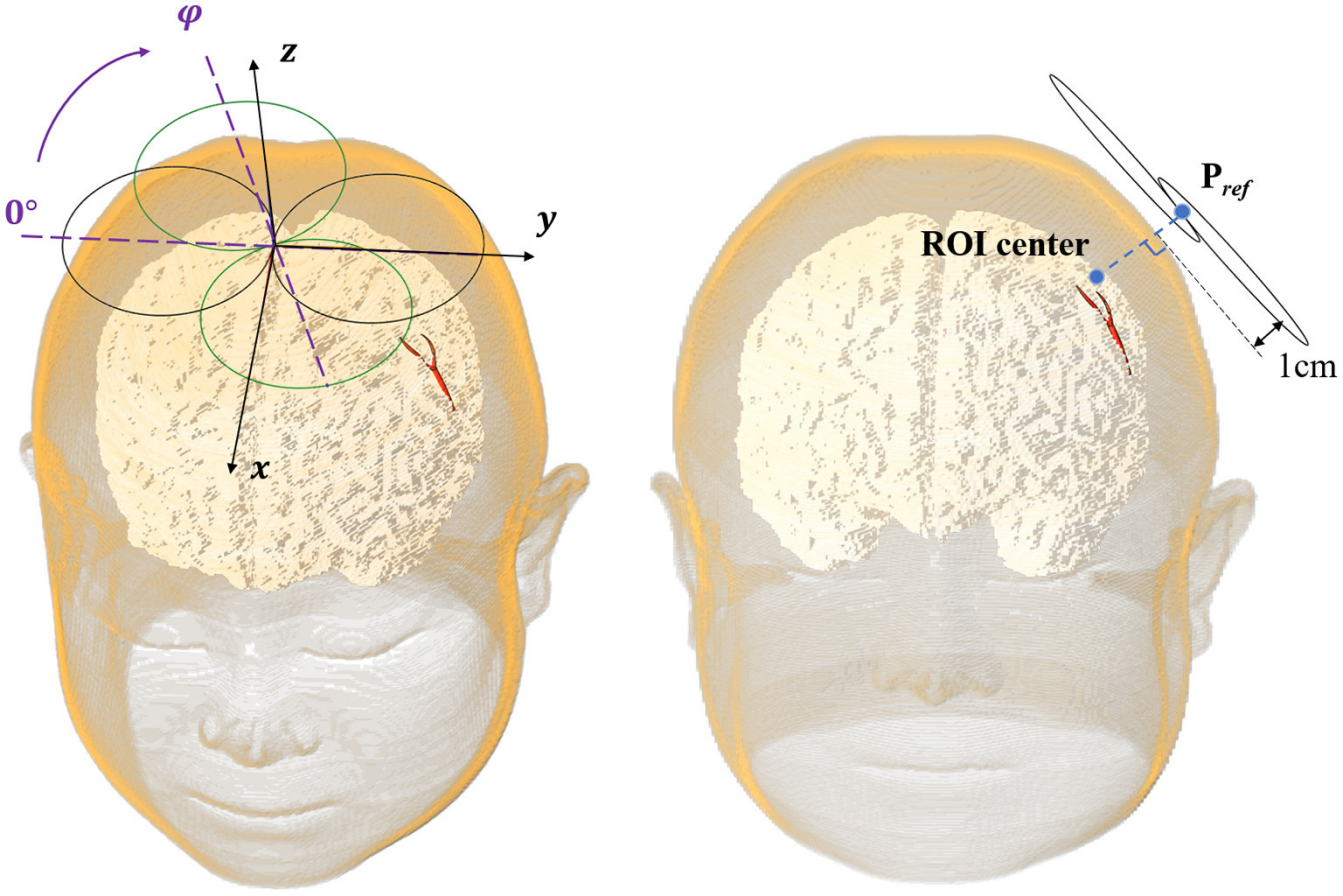
The eight-figure coil position.

In this study, we selected the *P*_*ref*_ (*x*_*ref*_, *y*_*ref*_, *z*_*ref*_, *and φ*_*ref*_) pointing out 1 cm from the lateral normal of the scalp above the center of the VOI as the clinical reference stimulation spot (Figure 3).

For comparing the E-field of the VOI with stimulation spot located in *P*_*ref*_ and *P*_*opti*_, the percentage improvement in the E-field intensity in the VOI (*E*_*improved*_), spatial distance optimization (*d*_*changed*_), and rotation angle optimization (φ_*changed*_) of coil was used as quantitative indexes to evaluate the optimization degree of the coil. A larger value of the index resulted in a greater optimization effect.


(4) $$\begin{array}{l} E_{improved}=\frac{\left(E_{opti}-E_{ref}\right)}{E_{ref}}\times 100\% \end{array}$$

*E*_*improved*_ reflects the relative improvement as a percentage of the E-field intensity during the coil positioning in comparison with the simulation at a reference spot. The larger the value, the higher the efficiency of the algorithm.


(5) $$\begin{array}{lll} d_{changed} & = & \sqrt{{\left(x_{opti}-x_{ref}\right)}^{2}+{\left(y_{opti}-y_{ref}\right)}^{2}+{\left(z_{opti}-z_{ref}\right)}^{2}} \end{array}$$

(6) $$\begin{array}{lll} \varphi_{changed} & = & \left|\varphi_{opti}-\varphi_{ref}\right| \end{array}$$

*d*_*changed*_ means Euclidean space distance between the optimal spot and the reference spot and the φ_*changed*_ is the difference between the angle of coil at the optimal spot and the reference spot. A large value of both means a different position.

For slight injury stroke lesions (<10 cm^3^) (48), a specific frequency stimulation is often operated on the affected hemispheres to maximize the activation of the undamaged brain functional connections and enhance the plasticity of the affected cortex; thus, accelerating functional recovery (49, 50).

In this study, the lesion area (2 × 2 cm) was surrounded by four 3 × 3 cm stimulation VOIs (Figure 2). Each VOI corresponded to a search domain (4 × 4 cm) for optimizing the coil position (31). The center of the search domain overlapped with the center of the corresponding VOI. The search domain covered all the VOIs and a part of the lesion and each VOI intersected with adjacent edges of the lesion and the other VOIs.

**Figure 4:**
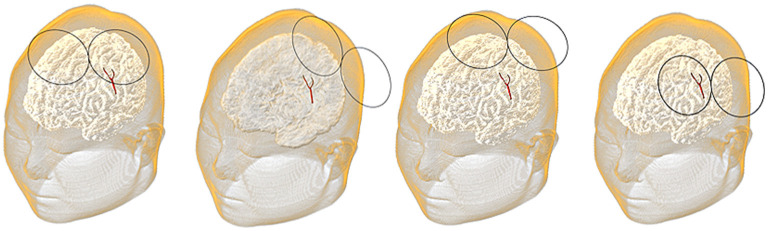
Coil positions in the voxel of interest (VOI) 1 to the VOI 4.

The position of the coil was determined by the spatial location (*x, y, and z*) of the coil center and its rotation angle φ around the z-axis (Figure 3). The coil plane was always kept parallel to the scalp. The position can be described as *P* (*x, y, z, and φ*) and the optimal coil position *P*_*opti*_ (*x*_*opti*_, *y*_*opti*_, *z*_*opti*_, *and φ*_*opti*_) contributed to the maximum average E-field intensity in VOI. Figure 4 shows the coil positions in the VOI 1 to VOI 4.

## Results

### Optimization Results of the E-Field

The E-fields in the VOIs of the ischemic stroke and hemorrhagic stroke models were optimized. Compared with the reference spot, the E-field optimization results are shown in Table 3.

**Table 3:** Optimization results of the electric fields (E-fields).

**Model**	***P*_*ref*_**	***P*_*opti*_**	***E*_*ref*_** **(V/m)**	***E*_*opti*_** **(V/m)**	***E*_*improved*_** **(%)**	***d*_*changed*_** **(mm)**	**φ_*changed*_** **(**°**)**
**Ischemic stroke**							
**The CVH female**							
VOI 1	(15.00, 40.00, 92.00, 45.00)	(19.00, 42.00, 90.00, 120.00)	1.75	1.92	9.59	4.90	75.00
VOI 2	(**–**37.00, 70.00, 77.00, 45.00)	(**–**41.00, 74.00, 70.00, 67.10)	1.50	1.55	3.34	9.00	22.10
VOI 3	(**–**22.00, 43.00, 97.00, 45.00)	(**–**20.00, 26.00, 105.0, 41.40)	1.57	1.81	15.32	18.00	3.60
VOI 4	(4.00, 77.00, 62.00, 45.00)	(12.00, 71.00, 67.00, 41.40)	1.78	1.85	3.82	11.18	3.60
Mean	/	/	1.65 ± 0.12	1.78 ± 0.14	8.02 ± 4.88	10.77 ± 4.74	26.08 ± 29.24
**The CVH male**							
VOI 1	(**–**36.50, 17.50, 60.50, 45.00)	(**–**46.50, 22.50, 55.50, 0.00)	1.89	2.57	16.99	12.20	45.00
VOI 2	(**–**70.50, **–**16.50, 47.50, 45.00)	(**–**73.50, **–**18.50, 46.50, 71.40)	2.50	2.67	6.82	3.70	26.40
VOI 3	(**–**37.50, **–**18.50, 65.50, 45.00)	(**–**34.50, **–**11.50, 66.50, 34.00)	2.20	2.36	7.19	7.70	11.00
VOI 4	(**–**81.50, 15.50, 27.50, 45.00)	(**–**77.50, 17.50, 30.50, 34.30)	2.50	2.77	10.89	33.38	10.70
Mean	/	/	2.27 ± 0.25	2.59 ± 0.15	10.47 ± 4.09	14.24 ± 11.45	23.28 ± 14.06
**Duke**							
VOI 1	(67.87, **–**32.95, 31.43, 45.00)	(66.88, **–**41.93, 26.44, 54.30)	2.05	2.22	8.13	10.30	9.30
VOI 2	(76.86, 16.97, 35.42, 45.00)	(78.85, 20.96, 33.42, 125.70)	2.71	2.80	3.60	4.90	80.70
VOI 3	(53.90, **–**5.99, 58.37, 45.00)	(67.87, **–**6.99, 43.40, 157.10)	2.08	2.19	5.21	20.50	67.90
VOI 4	(82.85, **–**12.98, 11.47, 45.00)	(81.85, **–**15.97, 12.47, 5.70)	2.32	2.39	3.10	3.30	39.30
Mean	/	/	2.29 ± 0.26	2.40 ± 0.24	5.01 ± 1.96	9.75 ± 6.73	49.30 ± 27.53
**Hemorrhagic stroke**							
**The CVH female**							
VOI 1	(15.00, 40.00, 92.00, 45.00)	(21.00, 40.00, 91.00, 172.90)	1.78	1.89	6.01	6.08	52.10
VOI 2	(**–**37.00, 70.00, 77.00, 45.00)	(**–**25.00, 58.00, 87.00, 137.10)	1.51	1.55	2.93	19.70	87.90
VOI 3	(**–**22.00, 43.00, 97.00, 45.00)	(**–**19.00, 27.00, 105.00, 47.10)	1.56	1.63	5.02	18.14	2.14
VOI 4	(4.00, 77.00, 62.00, 45.00)	(10.00, 69.00, 69.00, 47.10)	1.78	1.84	3.46	12.21	2.14
Mean	/	/	1.66 ± 0.12	1.73 ± 0.14	4.36 ± 1.23	14.03 ± 5.37	36.07 ± 36.21
**The CVH male**							
VOI 1	(**–**36.50, 17.50, 60.50, 45.00)	(**–**41.50, 26.50, 55.50, 174.30)	2.35	2.77	17.48	11.45	50.70
VOI 2	(**–**70.50, **–**16.50, 47.50, 45.00)	(**–**74.50, **–**11.50, 45.50, 65.71)	2.58	2.69	4.20	6.71	20.71
VOI 3	(**–**37.50, **–**18.50, 65.50, 45.00)	(**–**44.50, **–**22.50, 62.50, 11.43)	2.07	2.17	5.19	39.84	33.57
VOI 4	(**–**81.50, 15.50, 27.50, 45.00)	(**–**77.50, 17.50, 30.50, 17.14)	2.52	2.80	11.31	5.39	27.86
Mean	/	/	2.38 ± 0.20	2.61 ± 0.25	9.54 ± 5.33	15.84 ± 14.03	33.21 ± 11.08
**Duke**							
VOI 1	(67.87, **–**32.95, 31.43, 45.00)	(54.90, **–**44.93, 44.40, 20.00)	2.08	2.20	5.43	21.91	25.00
VOI 2	(76.86, 16.97, 35.42, 45.00)	(70.87, 22.96, 44.40, 120.00)	2.80	3.04	8.71	12.34	75.00
VOI 3	(53.90, **–**5.99, 58.37, 45.00)	(44.92, **–**6.99, 64.35, 177.10)	2.12	2.32	9.55	10.84	47.90
VOI 4	(82.85, **–**12.98, 11.47, 45.00)	(85.85, **–**12.98, **–**6.49, 11.43)	2.32	2.43	4.48	18.21	33.57
Mean	/	/	2.33 ± 0.29	2.50 ± 0.32	7.04 ± 2.13	15.83 ± 4.46	45.37 ± 18.96

**Figure 5:**
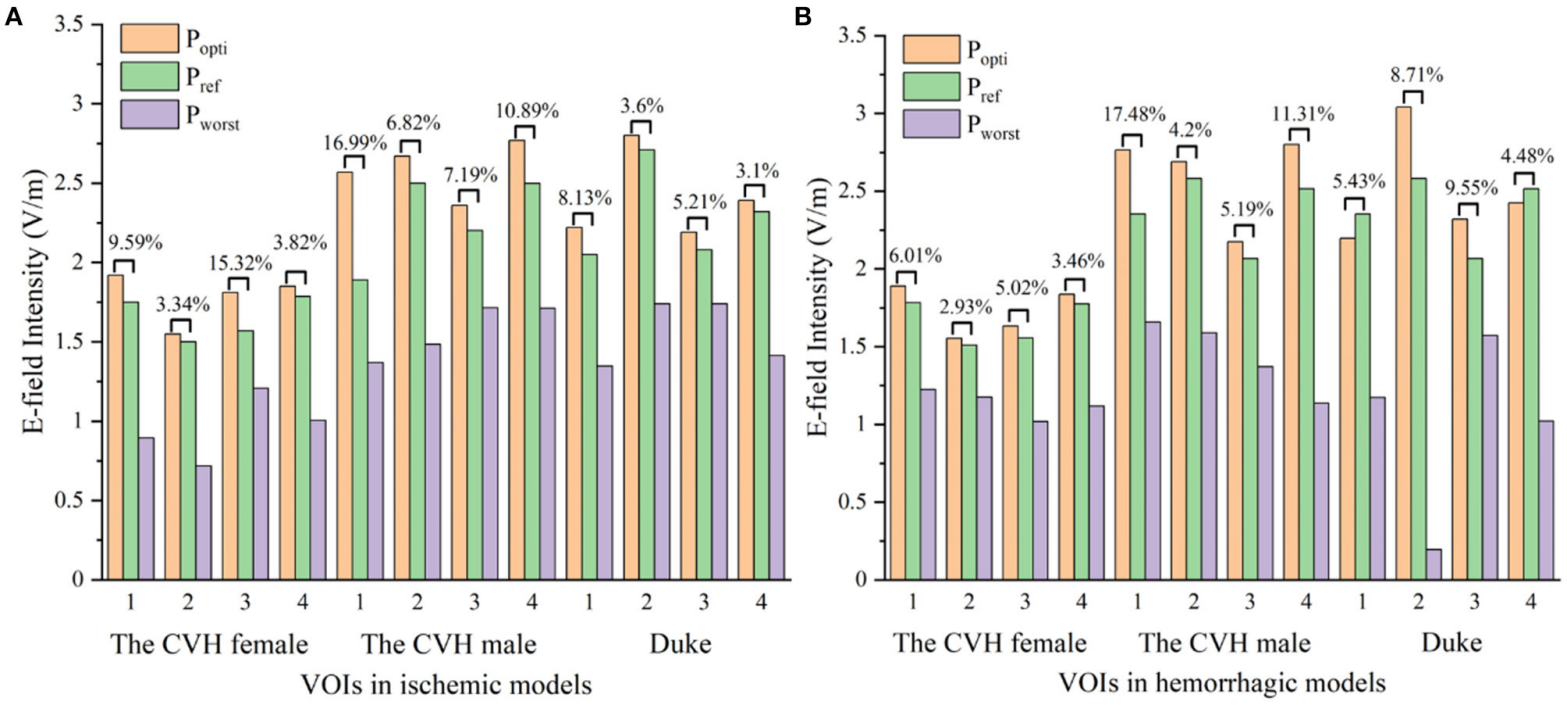
The electric fields (E-fields) at different stimulus spots in ischemic **(A)** and hemorrhagic **(B)** stroke models (*P*_*opti*_, *P*_*worst*_: coil stimulation spot in the search domain, where the VOI average E-field reached the peak and trough; *P*_*ref*_: clinical coil reference stimulation spot).

As can be seen from Table 3 and Figure 5, compared with *P*_*ref*_, the overall induced E-field in the VOI with the coil at *P*_*opti*_ significantly improved. The E-field improvement was lower in the Duke stroke model. The average E-field improvements in the VOIs ranged from 5.01 to 10.47% for ischemic stroke models and from 4.36 to 9.54% for hemorrhagic stroke models. There were about 66.7% of the VOIs that showed significant improvement in the E-field intensity (*E*_*improved*_ over 5%). During coil position optimization, the maximum spatial location change was 39.84 mm and the maximum rotation angle change was 87.9°.

To visually present the E-field enhancement in the VOIs, the distribution of the E-field in GM at two different stimulating positions (*P*_*opti*_ and *P*_*ref*_) in two stroke models was studied.

**Figure 6:**
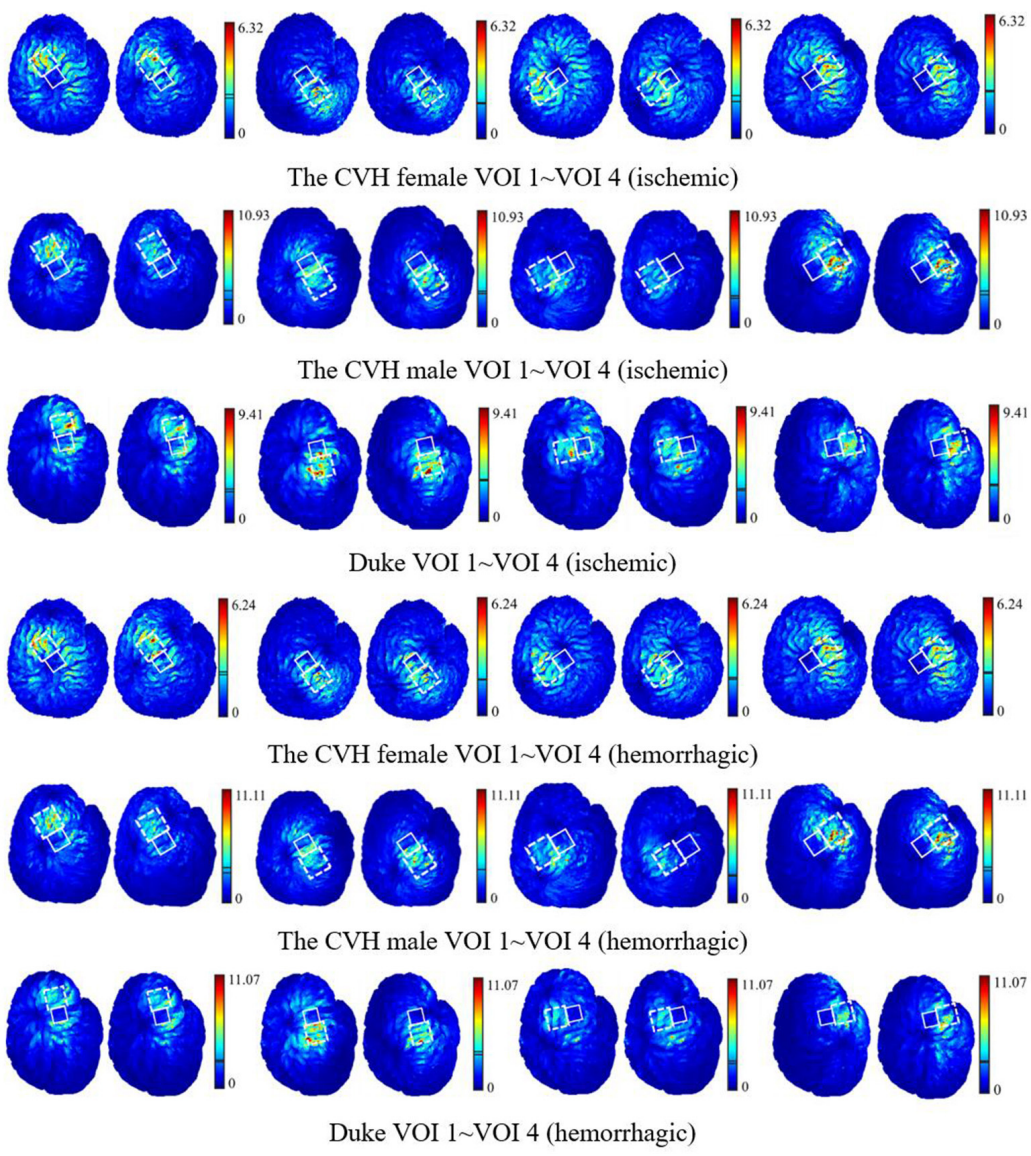
The E-field distribution of the VOIs with the coil at *P*_*opti*_ (left) and *P*_*ref*_ (right) [unit: V/m; the two lines in the linear color bar represent the average E-field intensity induced by *P*_*opti*_ (upper) and *P*_*ref*_ (lower)].

Figure 6 shows that all the focus centers of the eight-figure coil were in the VOIs and the average E-field intensity and the whole uniformity of the VOI were greatly improved after optimization of the coil position. The distribution of the E-field in the VOIs of the ischemic stroke model was consistent with that of the hemorrhagic stroke model. Furthermore, the improvement of the E-field intensity in the VOIs of the ischemic stroke model was higher.

**Figure 7:**
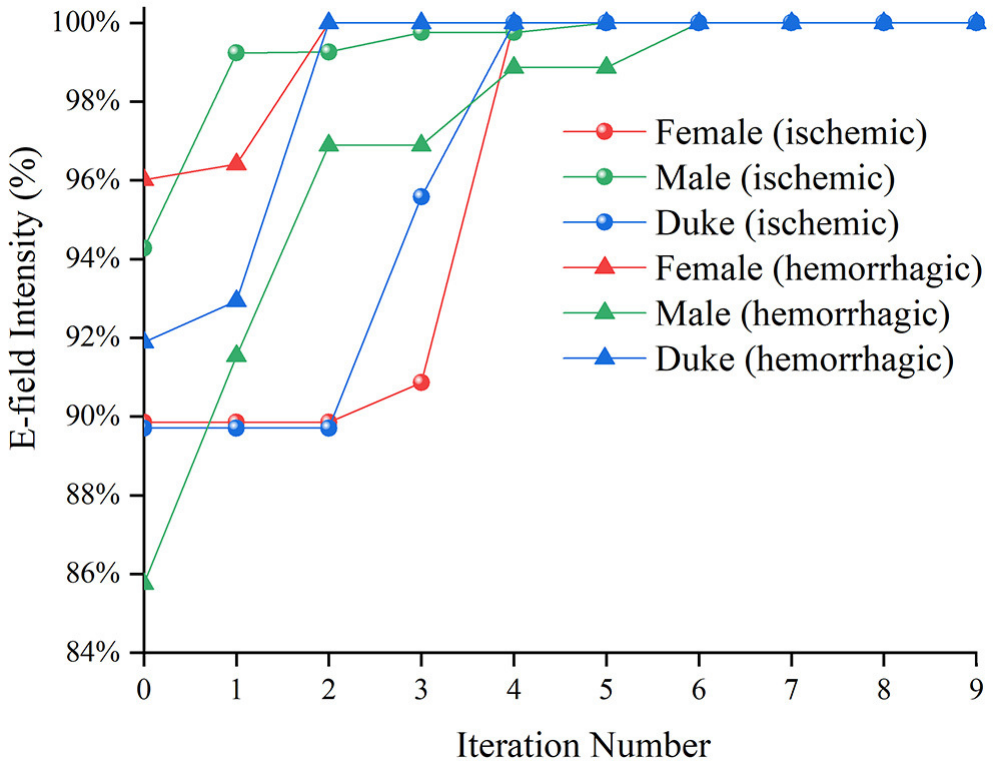
The average E-field intensity in the Chinese Visible Human (CVH) female (ischemic)-VOI 1 in every iteration.

The optimization iteration process showed that the E-field values were no longer updated after the seventh generation (Figure 7), implying that the algorithm converged effectively at the ninth generation. Therefore, an iteration number of 9 was selected to ensure the optimal position parameters in the coil search domain and each optimization of the model took 4.5–5.71 h.

### Validation of the Algorithm

To analyze the robustness of the algorithm, the CVH female (ischemic)-VOI 1 was selected to repeat the optimization process five times. The Δ*E*_*improve*_ below 1% was regarded as the no E-field improvement. The results are shown in Table 4.

**Table 4:** Algorithm repeatability verification.

**Test**	**E_***ref***_ (V/M)**	**E_***opti***_ (V/M)**	**E_***improve***_ (%)**
1	1.75	1.92	9.59
2	1.75	1.92	9.30
3	1.75	1.93	9.93
4	1.75	1.93	10.27
5	1.75	1.91	8.96
Mean ± SD	1.75 ± 0.00	1.92 ± 0.01	9.61 ± 0.46

The distribution of *E*_*opti*_ obtained using five repeated experiments was concentrated with a small fluctuation (SD = 0.01 V/m), indicating that our optimization algorithm could find the optimal position of the coil after nine iterations and exerted a stable improvement effect on the E-field of the VOI.

To verify the effectiveness of the algorithm in searching the optimal coil position in the search domain, a 6 × 6 mm ergodic search domain covering both the *P*_*ref*_ and *P*_*opti*_ was set. In the search domain, the distance steps in the *x-, y-*, and *z*-directions were set as 1 mm and each space point was set with 11 rotation angles (the rotation angle step was 11.25° and could cover 45°). The average E-field intensities in the VOIs under all the coil positions were calculated and compared with *P*_*opti*_.

**Figure 8:**
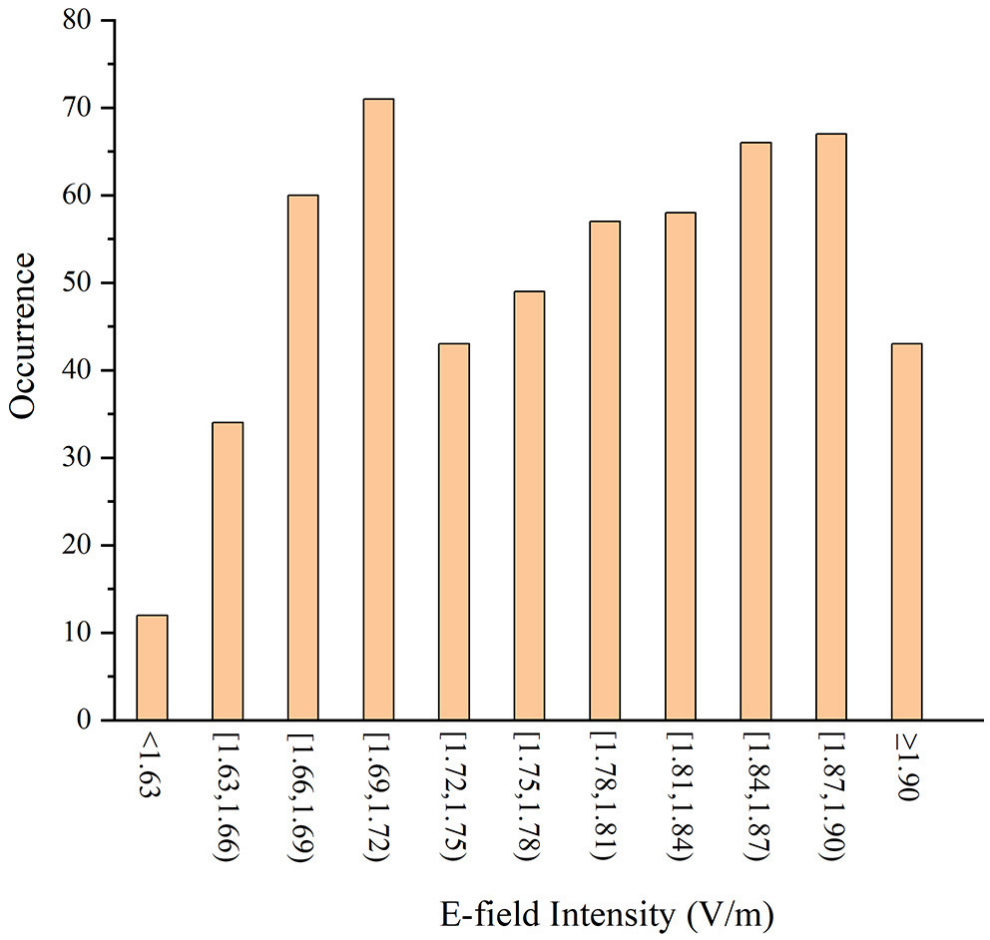
The average E-field intensity of the VOIs in different stimulus spots.

There are many coil positions that contribute to the higher average E-field intensity than the reference position (1.50 V/m) in the whole search domain for the same VOI (Figure 8). It can be concluded that the reference position is not very likely to be the optimal one due to the diversified structure of local cortical folds. The average E-field intensity in the VOI can reach the maximum value (1.92 V/m) when the coil stimulates at the optimal position given by the algorithm and this also confirms the accuracy of our algorithm.

## Discussion

Several differences exist in the structure of the local gyri among models and locations and, thus, the intensity and distribution of the induced E-field of the coil position in the different VOIs vary. For a better therapeutic effect, the E-field intensity in the VOI needs to be improved maximally. It is necessary to optimize the spatial location and angle of the coil for specific stimulation areas because there is no fixed paradigm for it.

The average E-field intensity in the VOIs of the CVH female was lower than that of others (Table 3), which was attributed to the specificity of the gyrus structure. The deeper and wider sulcus of the CVH female leads to greater filling of the cerebrospinal fluid (CSF) near the GM. As a result, the electromagnetic field travels a longer distance through the CSF, resulting in more energy attenuation. Therefore, the induced E-field intensity in the VOIs of female was lower than other models. The lesion in the CVH female was thinner than in others, i.e., more residual GM voxels were classified as the VOIs below the thin lesion (four edges of the lesion had about 2 mm intersection with each VOI). The presence of these GM voxels closer to the stimulation coil below the lesion resulted in an enhanced E-field. Therefore, the stimulation intensity can be appropriately reduced in the treatment of lesions with a light injury.

Figure 6 shows that when the coil was at *P*_*opti*_, the E-field distribution gradient in the VOI was significantly different due to the coil position and gyrus structure difference. The VOI E-field average of ischemic and hemorrhagic stroke models can be maximally improved by 16.99 and 17.48%, respectively, due to the optimization of the coil position (Figure 5). Although in a few models, the peak E-field of the VOI was higher when the coil was at *P*_*ref*_. A greater effect on the average value of the induced E-field and the field uniformity of the VOI was observed when the coil was at *P*_*opti*_, which could maximally activate the residual nerve around the lesion and improve the efficiency of stroke treatment. In addition, because the cerebral cortex has numerous folds, the optimal coil stimulation position, where the maximum overall E-field intensity in the VOIs could be acquired, tended to deviate directly over the VOI center (Table 3; Figure 6). When the scalp edge curve was steep, the distance optimization value increased significantly such as *d*_*changed*_ in the CVH female-VOI 3, the CVH male-VOI 1, and the Duke-VOI 1. The distance change fell within 20–30 mm, which was equivalent to the lesion size.

Previous study showed that induced currents pass perpendicularly through the local gyri to cause at least a 51% increase in the E-field (51), which means the coil at *P*_*ref*_ with 45° can cause the induced current to pass vertically into the central sulcus. Therefore, *P*_*ref*_ is generally considered as a good stimulus spot in the search domain (Table 3; Figure 5). However, gyri in the VOIs such as the VOI 1 and the VOI 2, which had a little distance from the central sulcus, were mostly not parallel with the central sulcus. Therefore, the eight-figure coil with a 45° angle could not let the currents be vertical to the CSF-GM boundary and resulted in the poor E-field improvement under stimulation of the coil at *P*_*ref*_. In addition, the optimization effect of the algorithm was obvious. Even for the VOI 3 and the VOI 4, which were closer to the central sulcus, the optimal stimulus angle was not 45° such as *P*_*ref*_. On average, the angle optimization was 35.55° and the coil angle optimization was up to 87.90° in the CVH female (hemorrhagic)-VOI 2. In conclusion, for the specificity of gyri, the clinical stimulation mode is insufficient to ensure that the overall intracranial microcurrent is vertically passed through the local gyri. To obtain safer and more effective treatment conditions, it is necessary to regulate the location and angle of the coil according to various cortical gyrus structures for different patients and different targeted areas in the clinics.

The coil position under the guidance of the optimization algorithm proposed in this study was optimal after nine iterations and exhibited excellent performance in five repeated experiments (Table 4). In addition, after position optimization, the average E-field and the distribution uniformity in the VOIs greatly improved. The optimization algorithm improved the E-field by 7.83 and 6.98% on average in the VOIs of the ischemic and hemorrhagic stroke models, respectively. The optimization algorithm improved the E-field up to 16.99% maximally for the ischemic stroke models and 17.48% maximally for the hemorrhagic stroke models. Furthermore, the enumeration method proved that the algorithm provided a global optimal coil position in the search domain (Figure 8).

This study result shows a significant improvement of the average E-field in the VOIs by the quantified coil positioning method, which reduces the positioning error caused by manual operation. By adopting GA, the electromagnetic response induced by pulse stimulus is maximized, the efficiency of magnetic stimulation can be improved, and the unexpected induced E-field in non-targeted brain area would also be reduced. As a result, this optimization process reduces the electromagnetic exposure time of patients and shortens the period of treatment to reduce the probability of the occurrence of complications. In addition, the efficacy of TMS is related to the gyrus structure of different brain regions between different patients. Yet, the proposed algorithm can be used to solve this problem by designing the clinical TMS stroke therapeutic schedule individually. The variable of the two stroke models can be abstracted as the electrical conductivity of the lesion. Therefore, our coil position optimization algorithm is robust in different diseases, with electrical conductivity changes in the local brain tissue, such as brain tumor treatment.

## Conclusion

Although the death rate of stroke is decreasing, its incidence has been continuously increasing worldwide and is higher in the younger population than in the elderly (1). To improve the efficiency of stroke rehabilitation treatment and provide patients with a more accurate and safe magnetic stimulation treatment in clinics, the ischemic stroke models and the hemorrhagic stroke models of the CVH female, the CVH male, and Duke were established. GA was applied to regulate the optimal coil location and rotation angle in a 4 × 4 cm search domain around the lesion, in which the rotation angle can be involved by the automatic search of coil spatial location within millimeter accuracy. Finally, the coil position is given in every VOI.

The proposed algorithm can be used to guide TMS coil positioning in clinical settings to achieve a more accurate TMS treatment. Contributing to the applied algorithm, the output energy of TMS can be freely dependent on clinicians to improve TMS stimulation efficiency and the dose of unnecessary region can be well-controlled; thus, the risk of electromagnetic exposure as well as the incidence of complications such as epilepsy can be lower, which is of great significance to public health and safety. Besides, benefitting from the robustness and repeatability of the algorithm in different races and strokes, our method can also be used in the plan of treatment for people of different races. This study gives a more accurate clinical quantitative treatment scheme and a proper stimulus dose plan for a safe and precise TMS treatment, which shows great prospect in stroke rehabilitation treatment. Furthermore, the proposed algorithm is also expected to be applied to TMS treatment of brain tumors in the future.

## Data Availability Statement

The original contributions presented in the study are included in the article/supplementary material, further inquiries can be directed to the corresponding author/s.

## Author Contributions

SL designed the model and realized all the three-dimensional (3D) models in this manuscript. HJ designed the algorithm to realize the optimized position search near the lesion. CL participated in the design of the model and the verification of location optimization and analyzed data. BH took part in the design of GA and the verification of data. PZ and WL provided ideas for the algorithm selection, model building, and electromagnetic field calculation and gave detailed guidance in the process of writing this manuscript. All the authors have read and agreed to the published version of the manuscript.

## Funding

This study was supported by the Science and Technology Project of State Administration for Market Regulation (2021MK152 and 2020MK149) and the Basic Scientific Research Funds of the National Institute of Metrology (China) (32-AKYZD2014–2).

## Conflict of Interest

HJ was employed by China Academy of Telecommunications Technology. The remaining authors declare that the research was conducted in the absence of any commercial or financial relationships that could be construed as a potential conflict of interest.

## Publisher's Note

All claims expressed in this article are solely those of the authors and do not necessarily represent those of their affiliated organizations, or those of the publisher, the editors and the reviewers. Any product that may be evaluated in this article, or claim that may be made by its manufacturer, is not guaranteed or endorsed by the publisher.
